# Our Virtual Tribe: Sustaining and Enhancing Community via Online Music Improvisation

**DOI:** 10.3389/fpsyg.2020.623640

**Published:** 2021-02-23

**Authors:** Raymond MacDonald, Robert Burke, Tia De Nora, Maria Sappho Donohue, Ross Birrell

**Affiliations:** ^1^School of Music, The University of Edinburgh, Edinburgh, United Kingdom; ^2^Sir Zelman Cowen School of Music, Monash University, Melbourne, VIC, Australia; ^3^Department of Sociology, Philosophy, and Anthropology, University of Exeter, Exeter, United Kingdom; ^4^Grieg Research School in Interdisciplinary Music Studies, University of Bergen, Bergen, Norway; ^5^Interactive Research in Music as Sound, Centre for Research in New Music, University of Huddersfield, Huddersfield, United Kingdom; ^6^The Glasgow School of Art, Glasgow, United Kingdom

**Keywords:** community, improvisation, virtual music, music therapy, wellbeing, music education, community music

## Abstract

This article documents experiences of Glasgow Improvisers Orchestra’s virtual, synchronous improvisation sessions during COVID-19 pandemic via interviews with 29 participants. Sessions included an international, gender balanced, and cross generational group of over 70 musicians all of whom were living under conditions of social distancing. All sessions were recorded using Zoom software. After 3 months of twice weekly improvisation sessions, 29 interviews with participants were undertaken, recorded, transcribed, and analyzed. Key themes include how the sessions provided opportunities for artistic development, enhanced mood, reduced feelings of isolation, and sustained and developed community. Particular attention is placed upon how improvisation as a universal, real time, social, and collaborative process facilitates interaction, allowing the technological affordances of software (latencies, sound quality, and gallery/speaker view) and hardware (laptop, tablet, instruments, microphones, headphones, and objects in room) to become emergent properties of artistic collaborations. The extent to which this process affects new perceptual and conceptual breakthroughs for practitioners is discussed as is the crucial and innovative relationship between audio and visual elements. Analysis of edited films of the sessions highlight artistic and theoretical and conceptual issues discussed. Emphasis is given to how the domestic environment merges with technologies to create *The Theatre of Home*.

## Introduction

This article is a multidisciplinary examination of how online synchronous improvisation can have beneficial effects for participants. The authors have primary expertise in 4 related, but distinct disciplines: Psychology, Music, Art and Sociology, and the resultant methods and analysis constitute a multidisciplinary dialogue between these different disciplines. The global (COVID-19) pandemic of 2020 necessitated various types of lockdown, social isolation, and physical distancing procedures to be put in place around the world. One consequence of these measures was an immediate and drastic reduction in social interactions. There is already empirical evidence highlighting that the negative psychological effects of the measures implemented to mitigate against COVID-19 are serious and related to social isolation [(Fancourt et al., 2020b; Lob et al., 2020; Zacher and Rudolph, 2020; COVID Social Study, 2020)^1^ and more recently the assembled literatures in COVID Minds, 2020]^2^. Therefore, the need for individuals to find ways to stay connected to family, friends, work colleagues and wider communities has become and, at time of writing, remains paramount.

Amongst the many strategies reported to help communities stay connected, music was utilized as a particularly potent form of communal activity. There were many reports in the international media of music being used. These included local communities chanting a song of support in Wuhan, Italians singing from their balconies in Sicily and all over Italy^3^ and a DJ playing dance music for the local community from his balcony in Glasgow, Scotland^4^. In the United Kingdom one particularly evocative and popular example of this use of music was the formation of a virtual choir where over 15,000 people were able to come to sing together as part of a project hosted by The BBC^5^. These vivid and anecdotal examples of music providing social support are supported by published research this a considerable and growing body of evidence highlighting how music can enhance health and wellbeing in both clinical and non-clinical contexts (MacDonald et al., 2012).

The crucial point for this current project is that, given these fundamental features, engagement with music has the potential to provide considerable emotional support and a means for connecting with friends and colleagues during turbulent times (MacDonald, 2021a). This is a focus of the current article. Another important point is that musicians are significantly affected socially and economically by the pandemic, with their livelihoods under threat in addition to the effects of physical distancing measures. Given this context the present study investigates this “double burden” of psychological and economic vulnerability.

While music of all kinds has demonstrated links to wellbeing, it may be that special claims can be made for improvisation, outlined below (MacDonald and Wilson, 2020). Viewed not only as a defining feature of jazz music, improvisation is now studied in universities and conservatoires around the world as a post idiomatic form of musical communication (Johnson, 1995; Dibley and Gayo, 2018; Onsman and Burke, 2019). Improvisation as an accessible, social, creative, and non-verbal process, distinct from other areas of musical activity provides an excellent context for health and wellbeing applications. As a real time spontaneous form of collaborative creativity it is open to the moment and thus pliable, malleable, and agile. Improvisation facilitates creative interaction and is fully accessible to anyone regardless of training or experience. While genre based improvisation (e.g., jazz, folk, and Carnatic) requires specific knowledge of conventions and techniques, non-idiomatic improvisation, that is improvisation that does not conform to any stylistic conventions or genre expectations, is fully accessible. MacDonald and Wilson (2020) provide a detailed account of techniques and further evidence to support this assertion.

Improvisation can afford a multitude of interactive possibilities (imitation, silent listening, and playful explorations, etc.). This is particularly important since the emphasis is on process and exploration rather than product. These features mean that improvisation also makes unique demands on cognition (multimodal real time decision making). Improvisation also enables the non-verbal articulation of thoughts and feelings that may otherwise be difficult to develop.

Improvisation is also a defining feature of music therapy since it can facilitate a clinical relationship between therapist and client (Wigram, 2004; Foubert, 2020; Foubert et al., 2020). Moreover, it offers a way of being or dwelling together when other communication modalities fail and one that overlaps in ecologically valid ways with what people do (or did) in their daily lives (Ansdell, 2014). Within a therapy context, improvisation can have benefits for specific groups, including aiding rehabilitation after neurological damage, enhancing communication skills for young people with autistic spectrum disorders, and reducing stress and anxiety. A number of benefits have been reported for specific populations as a result of improvisation and MacDonald and Wilson (2014) provide a review of published evidence.

Particular features of improvisation that may facilitate these developments include engaging both conscious and unconscious perceptual processes and facilitating the exploration of difficult or repressed emotions and memories. Improvisation can be emotionally engaging and help distract from recurrent negative thoughts (MacDonald and Wilson, 2014). Improvisation may also make particular demands on attentional and perceptual processes while engaging participants in a uniquely social and creative process (MacDonald and Wilson, 2020). Importantly, although improvisation is a fundamental aspect of music therapy practice, its basic processes remain similar outwith clinical contexts. It may therefore offer health benefits to those engaging in improvisation activities outside of explicitly healthcare situations. Furthermore, improvisation is also a fully accessible form of musical engagement that is not limited by pre-existing canonical notions of mastery and instrumental technique (MacDonald, 2021b). This is apparent in the breadth of documented improvisational approaches (Cornelius Cardew, 1974; Oliveros, 2005; Stevens et al., 2007; Ninh, 2010; Morris, 2012, 2017). This is further substantiated in the general oral tradition of the field – for example influential improvising singer Maggie Nicols’ often spoken phrase “creativity is a birthright,” is a perspective echoed by many players in the field while also being a core value to GIO as well^6^.

While there is no agreed definition of improvisation, for the purposes of this article improvisation is constructed as a social and collaborative non-idiomatic form of creativity that focuses on spontaneous, real-time interactions. In other words, a unique form of universally accessible, socially mediated, and collaborative creativity (MacDonald and Wilson, 2020). Of particular importance here is that these features allow many of the challenges of online music-making to be incorporated into ongoing musical interactions. For example, latency is often quoted as a considerable barrier to online music-making. Latency occurs due to the time taken for events to travel via the internet from one location to another. Much has been written about how synchronous musical interactions are influenced by latency issues and there is considerable debate regarding how best to overcome the challenge of performing music online “in time” (everyone synchronized to the same pulse) (Smith et al., 2020). However, an improvised approach to music-making allows latency to become an emergent feature of improvisational interactions. In summary, the real time process focused aspects of improvisation, combining its accessibly, social and collaborative features, make it an ideal process to utilize during online music making. Within this online environment the unpredictable nature of technology (hardware and software) necessitates the need to be dynamically responding to moment to moment changes and surprises.

An important feature of the many examples of music performances that appeared on the internet during the COVID epidemic tended to focus on pre-composed music. However, playing together in real-time is particularly difficult given the current technology available includes delays in processing and transmission, termed latency and defined above. To compensate for the “latency problem,” musicians very often have to record individually and separately with the whole piece being later reassembled in the editing stage. While this production process can create an illusion of musicians playing together in real-time, it is achieved only by isolating musicians acoustically – in other words, they cannot actually be together and co-produce real-time sound. By contrast, the use of improvisation may offer strategies for overcoming latency issues and thus facilitate real-time interactions between musicians.

Considered within a “music, health and wellbeing” perspective it seems paradoxical that online music making, which has potential to offer comfort and connection, has been probematized because of the predominant (and under-examined) preoccupation with presenting pre-recorded “finished” products. This “problem” can be redressed if we reconsider the “performance” vs. “exploration” perspectives. In the performance perspective, the material features of *Zoom* are dismissed or viewed as in need of being disciplined/minimized/eradicated/effaced. In the latter, a focus on the material ingredients of *Zoom* in terms of what they afford for “becoming” and as those affordances are tapped and embraced in ways that lead to an emergent “Zoomesphere” (defined below). This online music making – specifically music making using *Zoom* in combination with the act of improvisation – can then be seen simultaneously as a new and important context for health related musiking. It is a place and space for new understandings in social and identity work, and for new ideas and new approaches to collaborative creativity. It also contributes new aesthetic discoveries and developments in ways that lead to an enhanced and broadened concept of what music is and can become.

## Materials and Methods

This project focuses on the work of The Glasgow Improvisers Orchestra (GIO) a large improvisation ensemble with a flexible membership or approximately 25 individuals. GIO includes musicians from a diverse background with its members (current and past) having also performed with groups such as The Scottish National Jazz Orchestra, The BBC Scottish Symphony Orchestra, Franz Ferdinand, and numerous critically acclaimed folk and experimental groups. The group also collaborates with dancers, artists, filmmakers, and writers. Since its inception in 2002 GIO has enjoyed a high profile in the international experimental music scene and have collaborated with some of the world leading musicians in this area, including (George Lewis, Maggie Nicols, Evan Parker, and Joëlle Léandre). It has also been the subject of high profile critical attention (“One of the best large improvising ensembles in the world.” BBC Jazz on 3. “The Premier league of the European improvisation scene.” Sudeutsche Zeitung^7^). The group has released 11 CDs and numerous works have been composed for it and/or commissioned.

As a means of staying connected during lockdown measures GIO began online improvisation sessions. Musicians from other parts of the world also experiencing similar “lockdowns” were also invited to the zoom sessions. This international dimension is particularly important since it utilizes one of the unique affordances of online music making using *Zoom*; namely it transcends geography. In these sessions all participants around the world were in the same creative space with the same remit to create music together. Sessions lasted 2 hours and were facilitated and recorded using *Zoom* software. Participation grew to include over 70 cross-generational musicians from around the world which had an emphasis on gender balance of approximately the same number of female identifying and male participants and inclusive of gender non-conforming contributors. All participants were experiencing some form of government instructed lockdown as a result of Covid-19 pandemic. The principal musical process at the sessions was improvisation which was used as a means to facilitate the large ensemble improvisations. Following the sessions, a sample of 29 individuals who had participated in the sessions were interviewed in connection with their experiences. Ethical approval was obtained from the Edinburgh University ethics committee and all participants completed a consent form and took part in semi-structured interviews. All interviews were recorded on *Zoom* and fully transcribed.

The types of musical activities included in the session were wide ranging and there was an explicit acceptance that all musical gestures were welcomed. Conventional playing, e.g., improvisations based on pentatonic scales, could easily appear alongside the use of household objects used for percussive and/or conceptual reasons (e.g., marigold gloves). Often musical gestures merged with other modalities and the dynamic use of virtual backgrounds became an important feature of the emerging creative dialogue (see Supplementary Videos 1–3 for detailed examples of the types of improvisation involved and also see below for further detail regarding the improvisation sessions.

The transcribed interviews were analyzed using a thematic analysis framework in accordance with criteria outlined by Terry et al. (2017). This approach included the following stages: Familiarization (transcription and repeated reading), Coding (initial labeling and description of topics); Generating themes (identify patterns and salience in codes); Reviewing themes (refining themes, ensuring accuracy, and checking with data); Defining and naming themes (developing clear succinct titles and definitions with appropriate exemplars drawn from data). In addition, verbatim quotes were identified and collated as qualitative evidence (Corden and Sainsbury, 2006). The analysis sought to recognize the inescapably “messy” features of coding and classification (Law, 2004) – in many cases codes overlapped and ran into each other as will be discussed below. Each member of the research team listened and/or read each interview at least four times (in some cases many more times). In line with guidelines above, coding was an iterative, emergent process, where authors endeavored to allow participants’ views and experiences to guide the coding. Similarly, and in keeping with the ethos of GIO, the team has taken a deliberate decision to include as many quotes from participants as possible in an academic format so as to let the voices of participants be heard in their own words and to get broader detailed quotes in support of emergent themes. In this respect the coding also utilized a grounded theory perspective. No coding software was used as it was not deemed necessary (indeed manual management of coding was viewed by the team as a means of becoming intimately familiar with the content of each interview). There were over 6 rounds of coding.

In addition the authors participated in the music sessions and during the writing of this article they reflected on their experiences in the sessions and their own wellbeing in relation to GIO. Between them they represented divergent levels of experience and skill within the world of improvised music and this divergence afforded a “dual handed” ethnographic focus on sessions. On the one hand, MacDonald, Burke, and Donohue were all seasoned improvisers who enjoy international reputations as musicians. On the other hand, DeNora, entered the group as a researcher/participant and a beginner to the field (relearning an instrument after 40 years) which allowed W to reflect in particular on the ways in which the group culture fostered inclusion, regardless of level of expertise. Birrell, an established artist and film-maker, oversaw the recording and subsequent editing of the sessions.

A key aspect of this study is the utilization of the visual aspects of the sessions for analysis. All sessions were recorded for both sound and visual elements. Three extracts representing a dynamic range of the musical, social, and psychological aspects of the interactions were selected and edited and are available here: Supplementary Videos 1–3. These films were analyzed with the TIAALS, software developed as part of the Interactive Research in Music as Sound (IRiMaS) project^8^. Particular moments are highlighted below as being good exemplars of the conceptual issues discussed.

## Results

**FIGURE 1:**
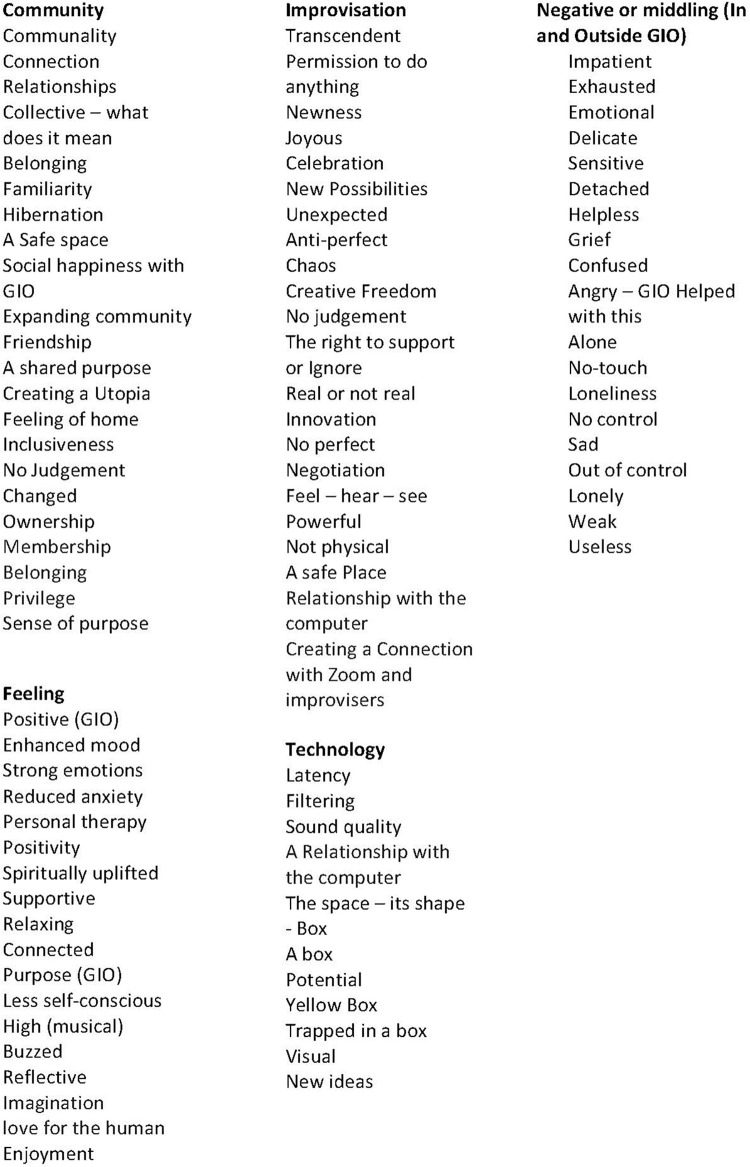
Emergent themes.

The first stage of analysis produced over 80 emergent themes and these are outlined in Figure 1.

**FIGURE 2:**
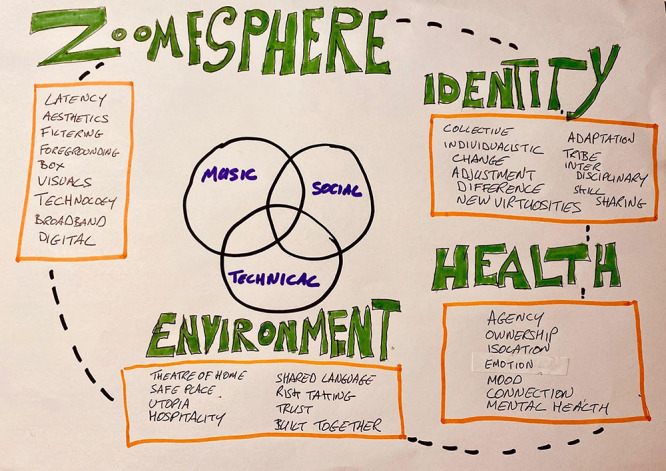
Thematic categories.

These themes were refined and further analyzed to produce three main thematic headings: Music, Social, and Technical. Statements referring to the musical aspects were placed within the Music category while comments regarding the wider psychological and social contexts of the sessions were categorized under Social. There was significant discussion around the technological features of the sessions, and these were resultantly coded within the technical category. Importantly, the analysis revealed considerable overlap between each of these broad definitions and the subheadings highlight how the musical, social, and technological aspects of the experience are inextricably linked. These, largely self-explanatory, categories helped provide a framework for four further broad themes: Identity, Health, Zoomesphere, and Environment as seen in Figure 2.

### Health

Physical and mental health issues were frequently raised by the participants. These observations referenced the negative effects of the pandemic along with the positiveness and elevation of the experiences of improvising in the regular GIO sessions. This was evidenced in the participant’s discussion around health issues in which they made frequent reference to the negative effects of the pandemic upon mental and physical health. One of these centered on grief, pain and distress:

It was a bit like, fuck. It’s like, we can’t play … we can’t be musicians. How do we like function in this life? (Participant 1).

I’d had this period of grief, which was kind of really unusual for me (Participant 2).

I thought it was too painful to imagine that I might not, you know, like that kind of huge orchestral sound, I might not get to do that again. So, I kind of shut down a bit. I didn’t like it… playing would remind me of all the things I couldn’t do, until I started doing the GIO thing (Participant 3).

I was overwhelmed by grief. I was having a lot of sleepless nights. I think you guys have probably had the same but just really weird dreams. Difficult things, right? (Participant 4).

All the quotes above highlight the explicitly negative effects of lockdown social distancing on health. However, there was an overarching sense that involvement with GIO helped address these psychological issues:

And so yeah, psychologically, it was. It was a benefit, I would say it was beneficial to my mental health during the look down, for sure (Participant 5).

GIO has been a very important part of nourishing me in a way that is, you know, lifting my spirits enough that I don’t default to addiction (Participant 6).

I had a lot of time to practice, but, so, I enjoyed the practising, but I felt alone. So I feel isolated. So, I need to, play, with, other musicians. So. Yeah. So Zoom session is very… I can overcome this feeling from the Zoom session…[I] got a sense of security. Thanks to the regular weekly sessions, I realized once again, how important music is to me (Participant 7).

Helped me to kind of kind of build on the core again and just and it’s a little bit like hibernating, I think, you know, you kind of like eat enough of that and you can sort of last a little bit longer by yourself for a while (Participant 21).

This is a very good method for me to not only mend my loneliness (Participant 23).

They give me something to do. And they give me something to look forward to. And they give me goals (Participant 24).

There were also many comments relating to how participation in the sessions resulted in beneficial effects. The first of these was connection:

I was seeing through lock down that feeling of being disconnected. Usually, my friendship circle or my social circle were all connected to the music scene. And so being able to engage with people in that way and still make music and connect with them, and just talk about how we’re all doing in the sessions and interact and having a laugh. All that was incredibly beneficial. And I noticed that it was something that I had, I noticed that other people maybe didn’t have (Participant 5).

I think that probably one of the things I was most scared of when the lockdown happened was that, obviously, with moving out of the city, moving to a different city, but knowing even if I went back to the city, I couldn’t just call up friends and meet with them, or I couldn’t see you all once a month. I just had a big fear of losing these connections. Even if, you know, we were allowed back in the Centre of Contemporary Arts within a matter of months, I know that this kind of connection and network is really essential to my wellbeing (Participant 9).

Yes, I feel a bit terrible these days … It feels like I’m connected to something that I belong to someone/thing … so having a group that I can feel that I belong to, and especially during this lockdown is just like, – like a miracle really. And I can sense that people are also on the same level or on the same path in a way. So it’s definitely like super important (Participant 11).

I don’t think I would have survived pandemic without this group. Without Raymond and GIO. I don’t think I would be feeling this good (Participant 4).

…They’ve managed to create a space of connection. And connection is something that every person needs (Participant 8).

So yeah. Benefits are, yeah, intense emotion induction during a time that a lot of our emotional baseline is just anxiety and uncertainty. Just maintaining connection, human connection, and friendships (Participant 9).

…The main thing for me was just connecting to the community, again, like and just having that sort of borders dissolve straightaway in seeing people from, you know from the year United States, United Kingdom, and Australia, and just being able to say hi, and then hear them hear them play again (Participant 21).

I live in isolation with my MS (Multiple Sclerosis) which I have had for 20 years and so this gives me connection (Participant 22).

These thematic strands reveal important aspects of the sessions linking musical engagement with psychological wellbeing. While each quote conveys subtly different meanings, they all emphasize that the online sessions helped participants feel connected to a wider community of supportive colleagues and friends. There is evidence that this type of psychological support is important. For example, high levels of social support and reducing stress are linked to more effective coping strategies (Curtis et al., 2004). While peer group social support and feelings of freedom through creative expression have been linked to increased hedonic tone and reduction in anxiety (O’Callaghan, 2001). Additionally, social support, feelings of connectivity, positive psychosocial moments and opportunities for creative engagement, play an important role in quality of life for clients in improvisational based music therapy (Aldridge, 1991). The key points here are that lockdown had a detrimental effect on participants’ health and that the online sessions helped ameliorate these negative effects by providing social support through feelings of being connected within a supportive community.

### Mood

Another consistent theme reported was the beneficial effect of a positive mood change. Many of the participants reported that the GIO experience resulted in a feeling of being simply elevated and “in a good mood” after the sessions.

Even if I wasn’t sure if I was in the mood for it, I always kind of came away with a really positive energy from it as well. Well, I think a lot of people felt that. And so yeah, that that was the main, the main thing was just that feeling of being connected to other people when we were so disconnected (Participant 5).

It’s just been really nice to see, you know, yeah, I’ve always been in a good mood afterward. It has always lifted my mood….. It has helped. It helped my mood, I guess I think, not that I was in a particularly bad mood when I started, but it was something that I could look forward to. And then when I did it, I felt, you know, much better than that. So, so yeah, I would say that it’s helped in that sense (Participant 10).

While others spoke about the experience as cathartic and empowering: explicitly recognizing the link between improvisational activities and health.

I’ve looked forward to it, you know, I feel like it’s like a kind of an internal massage for your brain almost, and being able to get to that focus state, that state of flow is the same. Really cool, because it’s, you know, you can’t call it that you’re talking about sleep, like I think people’s sleep has suffered. I think concentration has suffered. It’s hard to focus. I mean, or it has been harder to focus, but I think that finding the focus through music, and then you realize an hour’s gone by, and then you’re like, wow, you know, so even that, having to do that, and it’s been really, really is beneficial for me (Participant 1).

These feelings of empowerment and positive moods also affected an enhanced sense of creativity:

But yeah, actually having these, these sessions to regularly and immediately make music, but also be an agent in the, the sort of stimuli for that music, you know, maybe more of an agent in the compositional process. Has made me feel really empowered (Participant 9).

Additional health benefits seemed to stem from a strong sense of feeling accepted by the group which participants reported producing a sense of belonging during extreme times of isolation.

I think it gave me what is it this sense of belonging (Participant 11).

So, being invited to the GIO sessions, you know, it has felt like a validation of me Whatever it is, socially or musically. And so, I have felt that I felt a sense of belonging (Participant 12).

Just to remind you that you’re not alone, you know, that you that there are people out there that want to relate to you and your part of a community. You know, we’re an extended family really (Participant 6).

Significantly, many observed that the space minimized judgments from others of their playing as well as how they judged themselves compared to previous in-person experiences. Australian improviser Participant 13 talked about the online creative space where a freedom from previous conventional improvisational spaces allowed unexplored interdisciplinary expressions and experimental choices to be made:

I found it all very liberating, you know, in the fact you’d have your own space, and it’s totally non-judgmental,… no judgment, no expectation, no sort of pressure that you could do anything wrong (Participant 13).

So there isn’t the sense of I’ve come to a place – What do people think of me? I feel safe with GIO anyway but there was that sense of even another layer of “ach” I’m at home I can put my feet up. I can just flash something on the screen, I can bring a teddy bear, or…I can play with colors. There isn’t a sense of it has to have meaning! It has to be important! I think taking that pressure off, it creates something quite magical (Participant 6).

This non-judgmental culture further enhanced participants’ willingness to take risks within the space, to offer things to the group that might otherwise have seemed outside the frame of what could count as appropriate. For example:

I think like it was certainly delicate and sensitive. Um, but I think there was a huge amount of trust there and huge amount of kind of warm support I mean, I personally found it very emotional really too, you know, when you some you got, I mean, some of the people there I know nothing about – (Participant 13).

In summary, these extracts all highlight the positive effects of the session on mood and in particular show participants awareness of their own mood management strategies. The notion of “social prescribing,” or referral of a patient, usually by a link worker, to a community asset or activity such as a sports club or choir, is currently generating growing interest (Fancourt and Finn, 2020; Mahase, 2020). While the effect of the GIO sessions in terms of mood shift can be understood through the social prescription lens, what happened here was also different. Members were engaged in “self-prescribing” and participation in the sessions can be understood as part of their lay-activity of self-care, and in ways that resulted in improved mental health and wellbeing, a finding that underlines the importance of processes and modes of engagement in the arts/mental health equation (Fancourt et al., 2020a), in this case the role of member-expertise and co-creation of the cultural practices at stake.

In relation to these modes and processes within GIO, it is worth reflecting on why it was that the tenor of participants’ responses was so overwhelmingly positive about the GIO sessions in relation to mental health. We believe this was an effect of (a) self-selection (all members chose to join the sessions), (b) familiarity with the people and the practices and all being in the same boat in terms of learning the techniques of zoom sessions and zoom’s constraints/affordances (including the opportunity to engage creatively with zoom so as to work, as improvisers, with those constraints/affordances, (c) the pre-existing culture within GIO of hospitality which was strongly linked to the core ethos of mutual support, and (d) the alternative – if GIO sessions were abandoned or if overt-conflict were to ensure there would be a return to social isolation. In short, GIO’s pre-existing culture afforded social support and the Covid-19 restrictions intensified the need members felt for a form of group support and companionship, musically configured. It is worth adding here that the weekly sessions, attended by all five authors, confirm unambiguously the convivial and “upbeat” quality of the sessions to which the interviews refer.

### Identity

Closely related to health were issues of identity which appeared to grow from the connection and continual contact with a digital online community. Identity has become one of the most discussed and problematized topics within contemporary society (MacDonald et al., 2017). Social changes taking place during the pandemic bring resultant implications for identity processes, and vice versa (Jetten et al., 2020) and this was clearly evident in the interviews. At times these developments were related to individuals’ sense of their own situations evolving, whilst the identity changes clearly related to the emergent group process in GIO sessions. For a number of participants their practice changed over the course of the sessions with discussion of creative evolutions. These developments consequently lead to changed notions of professional identity (Isenberg-Grzeda, 1988).

…We are developing a new way of being creative as we’re doing it (Participant 1).It’s really redefined my identity again, it is a reflection to what’s going on now and during this time if I cannot perform onstage, so what. I’m supposed to stop working? Stop singing? Stop doing this stop doing that? No! So I need to redefine what is art for me, what is what kind of interaction, what kind of way I want to reach to this world (Participant 14).

For some people, the urge to be creative and to be part of a community is possibly more fundamental than the urge to be an instrumentalist. Some comments expressed a basic human need to be creative (Burnard, 2012).

Finding my GIO tribe has been amazing. But that’s not always you know, that was okay for me living because I moved to Glasgow, which is one of the most amazing creative and kind of permissive in a creative way. Cities you could live in. You know, it’s like, I always think it’s like, nobody You can do whatever you like in Glasgow and the kind of the combination of nobody really cares. And also, but they’re willing to let you have a go, some kind of combination of that is just I think it’s why there’s so much, you know, art and music and just, it’s respected, but also know, nobody’s going to really ask me if I didn’t get all the things. So if I put if I hadn’t moved to Glasgow, there’s no way I would have found that tribe. I don’t think in Cork I although maybe there are people here now, but I’m not sure I’ve ever come across them. So that is that that’s where the virtual tribes are going to are going to really come in for people who aren’t as lucky as I am to live in Glasgow. And to be able to sit in a room with people like Raymond, you know, that’s where, and you’re going to have people and Raymond as good example, who are going to be the ones to reach out to make and networks across the world that then you know, the little kind of local communities and we’ll be joined up by people like Raymond and yourself and you know, Who do and get to spread the word around to this? And I, I just think that yeah, it’s just that finding your tribe thing, it’s the same I feel the exact same way about the, the Chamber Orchestra I play with like, it was, it’s just like, in you making music with your friends or your more, or people that you feel are accepting of you. So you’re more relaxed, you can do more (Participant 15).

As posed by Maffesoli (1996); Hetherington (1998), and Bennett (1999), the idea of the tribe captures the temporal and spatial fluidity of belonging and attachment in modern times because it abstracts identity from its traditional locations (gender, class, region, occupation, education, culture, and sub-culture). By contrast, identities emerge as individuals move between site-specific cultural scenes associated with persona, practices, and stances and the basis of social order within these spaces is empathic, based upon ambience, emotion and aesthetic practice (Maffesoli, 1996). At the same time, the tribal impulse is underpinned by, as Hetherington (1998) suggests, the strong desire to belong, understood as a response to increasing social and cultural disruption and fragmentation:

Oh, you have to find your tribe, it really resonated with me because that’s what being a musician is (Participant 3).

That whole thing of bringing everybody together creative people together, to, you know, to improvise and to feel like they belong. We know Yeah, like minded you know (Participant 16).

I think of GIO in my head, I just think of it as this amazingly warm family…very powerful thing. Powerful thing. Good juju (Participant 10).

The impulse to belong to a tribe is in other words a survival strategy and a part of what might be seen as a tacit salutogenic practice. This is because the tribe can offer an affective haven or place/space of and for expression, comfort, pleasure, recreation, and play. As such the tribe is also is a site of mutual performance (Bennett, 1999). As we can see in the quote from Participant 15, the tribe can be understood as a “community of feeling” (Hetherington, 1998) or “a certain ambience” (Maffesoli, 1996, p. 98) involving sensibility (Onsman and Burke, 2019), aesthetic orientation and affinity with like-minded others. In this sense, tribal belonging is a source of sustenance, wellbeing, and identity validation. It is a space for health promotion. Of interest then is what the space of *Zoom* affords in terms of wellbeing in these turbulent, COVID times and how that space can be appropriated and rendered into a kind of “home from home” (‘moved into’) so as to preserve connection when the traditional physical spaces for tribal congregation are prohibited or, as with Covid-19, temporarily out of bounds. Identity-transformation in and through music was a crucial feature of this “moving in,” and in particular for this international group where it served as a communal language and promoted inclusion.

Because we really should have our language to communicate…[music] is actually much easier for me, talking English is much harder. The music, our language, we can discuss/talk. I mean, exchange the feeling, so somehow doesn’t matter where you are. In United Kingdom, in Japan, in Europe or in America. Everybody. Most of us had to stay [in] our place. Not go out. And we didn’t know what’s going on. And you know, facing…COVID. So it’s like sharing feelings and making music. I think even just doing that, just two of those things. Makes us all feel, I mean, very happy (Participant 17).

The focus on the creation of tribal connections through the medium of an online platform, moreover, highlights an important theme of this article: the interpenetration of the “actual” and the “virtual’. That theme in turn illuminates the particular qualities of the Zoomespheric, virtual space for GIO members. Cultural geographers have considered how, in Shields’ words, “the virtual is real without being actual and ideal without being abstract” (Shields, 1999, p. 3). In other words, identities are always hybrid; they emerge in relation to materials and practices and in ways that shuttle back and forth between, and mutually constitute image, dream, virtual, material, possible, and actual (DeNora, 2014). In the case of GIO online, the specific features and affordances of the Zoomesphere, understood as a space, were as participants described; critical for the creation of new modes and practices of belonging and experiencing identity – personal and social. Furthermore, they emerged out of what, initially, were perceived as constraints posed by the *Zoom* software and in ways that expressly link that technology, and its affordances, to the previously discussed themes of identity, mood and health.

### Zoomesphere/Environment/Space

While there is specific networked music production software (Jacktrip^9^, JamKazam^10^), it is of note that all GIO meetings opted for a visual conferencing platform over the musical platforms. *Zoom* was chosen specifically for its inclusivity (easy to access, no technical knowledge needed). As adaptations to the Zoomesphere proliferated this new space became, as we call it, “moved into’.

A space is itself a hybrid of material, conceptual, imagined and practical. It is, as Wierzbicki and Nakamori (2005). have said, “a place and space in which knowledge is shared, created and used, including physical space (offices, buildings), virtual space (computer network services), and mental space (experiences, ideas, emotions) – a place and environment in which creative activity can be performed”. A common thread highlighted in the interviews was the notion of the musicians being part of a virtual creative space. This virtual creative space (Zoomesphere) included the physical room where the musicians performed (a room in their home), the computer screen and the *Zoom* software where each musician had their own box: their space amongst the larger ensemble space of stacked spaces on the screen. Within the structure of stacked boxes on the screen, each music improviser was afforded agency to create and experiment both visually and with sound. Within this stacked structure, each box can simultaneously be perceived as an “individual stage” and as one part of a wider stage or scene. Through the shared space and from within the individual boxes (and thus the overall Zoomesphere) they can be seen to be involved in a constant and co-creative configuring, negotiating, and reconfiguring of the Zoomesphere. In this sense, the GIO improvisatory Zoomesphere can be seen as an exemplary case of democratic co-creation and important in the observed wellbeing effects.

I really loved the way GIO was set up. I love the egalitarian structure. And the fact that they interrogate that structure in a very rigorous way. And they were able to translate that into the Zoom format. That was really interesting. I thought artistically (Participant 8).

The visual democracy and flattened space that *Zoom* affords, draws to the fore the visual features of what transpires during a session which accords each participant equal space. The individual/social affordance of the boxes within the box let individuals have “equal space” (visually speaking) for furnishing what happens in the “bigger box” (the total of the individual boxes) and that this “affordance,” combined with the audio-constraints (can’t hear all at once, latency) drew the visual features of improvisation to the fore in a new way. While much has been written regarding how recent advances in community music and music education have fostered more egalitarian and democratic types of musical engagement, the type of democracy we refer here is specifically concerned with the technological affordances of working within the Zoom context. All participants occupy equal space on the screen and this situation is quite different from in-person music making where hierarchies are often explicitly conveyed in how a group is spatially organized on stage. For example conductor, lead singer, and soloist etc., at the front and drummer, percussionist backing singers at the front.

The theme of reimagining is significant here, as it draws into relief an important feature of our understanding of space as mental space (Fauconnier, 1994). Fauconnier proposed the term “mental spaces” by which he meant cognitive structures relating to non-reality through individual conceptualizations and ideas and their properties. The notion of mental spaces is intrinsic to an understanding of what a virtual space is through the GIO online improvisational experience. Many of the interviews support the argument that the virtual space took on an expanded meaning to include Fauconnier’s term mental space; a bricolage of the physical space, affordances, and mental space that make up the online improvisational creative space: the Zoomesphere.

There’s something very intimate about the fact that we are using our devices. We are using our phones. We are using our laptops: it’s something that is ours. So we have a kind of attachment to it to this, it’s an object, but it’s our object. So we are objectivizing the object itself. And we are seeing through our lens, something that is outside. So it’s like there’s something very rationalism like kind of approach of, you know, not even Cartesian approach but you know, just kind of an idea of the consciousness, like we are here and we are actually observing ourselves all the time. Because ourselves are always in the screen somehow. So while I have this phone and I’m going to an installation, that is using augmented reality, and it’s my phone, I know how to control it. I am the one who leads it. I am in charge. I’m god. I’m going to say it like that in a way. So while I’m taking this camera and opening it, first of all, it’s very, very intimate, because I’m using my phone. And then I’m starting to scroll it into the room and finding all kinds of clues inside of it. So I am creating my narrative. I’m creating my art form; I’m creating what’s happening. And it wasn’t before. And I also think that it’s also happening on *Zoom* (Participant 14).

Here, Participant 14 alludes to how entering into the Zoomesphere is entering into an “immersive space,” one that affords “new way[s] of thinking” that in turn are capable of enhancing or augmenting realities, which is in effect also about altering realities and with them consciousness. Importantly, the Zoomisphere offered participants a “new” space to relate and be self-aware. A space for inhabiting at a time when, during lockdown, the “real” space was characterized by enforced confinement within the “box” of the domestic environment and the requirement to “stay at home’.

Sometimes the loneliness of the pandemic felt more alone because you did realise, you’re trapped in a box (Participant 4).

The confinement in turn offered a contrast structure against which to perceive and experience the Zoomesphere as, alternately, a liberating space and a space of freedom:

It feels very free and open and, and I don’t feel limited in my own expression (Participant 12).

Some identified the Zoomesphere as a place that took them away from the confinement and restrictive nature of their lives to a space where they wanted to be.

So in a sense, it’s a little utopia that we enter in from time to time … in a way that may sound a little bit corny. That’s a world we want to live [in]. We don’t want to spend 24 h a day making music on Zoom, but we want to be in a world that everybody has support for each other, everybody is equal, and they respect everybody for what they do (Participant 18).

The Zoomesphere was also a space where the musicians reflected on what was happening in their lives while being in a “lockdown” through the pandemic. At a time, globally, when live music in venues closed, many musicians entered a type of musical depression as a result of an overarching change to the way they lived their lives. The Zoomesphere space gave the musicians opportunities emotionally to release themselves and as such it served as a kind of refuge or “music asylum” (DeNora, 2013): a retreat from the cares and stresses posed by the exigencies of daily life under Covid-19.

It was helpful to my mental health… there was no stress (Participant 3).

Once I settled into it, I found it all very liberating, you know, in the fact you’d have your own space, and it’s totally non-judgmental. I think that was another really important factor (Participant 13).

I don’t think I [would have] survived pandemic without this group. Without Raymond and GIO. I don’t think I would be feeling this good (Participant 4).

I was always constantly mentally, emotionally physically, and just doing everything I could to make sure I could be there at the next session and just listen and feel it and then respond and then create something new (Participant 4).

But, as the last comment from Participant 4 illustrates, the Zoomesphere offered something more substantial than mere retreat as the format of online collaborative improvisation also afforded the development of new creative, and social, practices. It was, in other words, a space that could be “furnished” with new affordances and resources for wellbeing (DeNora, 2013). For example, participants could project new content (and new versions of themselves) into the public space of the boxes. Furthermore, the medium for this transcendence was, as participants explained, a new sonic and aesthetic practice that made use of what the Zoomesphere afforded. Moreover, the perception of “space” took on new dimensions and brought with its creative forms of relating, making music together, and re-perceiving/re-conceptualizing the constituent features of freedom and/or constraint.

There was much discussion around conventional approaches to the ensemble versus the Zoomesphere: the sound that the musicians made in their room and how this translated to their contribution to the ensemble and sonic environment.

Making people respect each other’s musical space like really listening, listening and responding, leaving space (Participant 5).

The musicians questioned why they played music and reflected on how they felt when playing music in a traditional setting (concerts and rehearsals) compared to the online space.

I have felt, you know, I’ve come out and I felt really, really happy. I’ve looked forward to it, you know, I feel like it’s like a kind of an internal massage for your brain almost, you know that kind of, yeah. able to get to that focus state that sense that state of flow is same (Participant 1).

### Moving Into the Theatre of Home

The climate of trust that the Zoomesphere created was characterized by a kind of hospitality and willingness to share features of the home environment: views of self, including new looks, costumes, make-up, masks and objects through the medium of this newly adopted digital stage. Young children, babies and pets frequently entered the “performance space”. This necessitated a creation of a shared new artistic practice which has been identified to augment audio and visual aesthetical nuances, mediated via the agential implications of *Zoom* as a new improvisation technique.

So there’s an emergent form. You know, there’s an emergent form, but it’s kind of mediated through the technology. I mean, all the emergent forms are mediated through technology but…in this case [it] was *Zoom*. The frame set the form…[of] what we were going to do (Participant 10).

At first I cannot [feel] comfortable with the front of the iPhone. But now I can relax. So Maybe I can make more much more good music, I hope (Participant 26).

I really embraced the fact that the software was being so selective but it was random…I felt that that was almost like a true free improvisation… (Participant 29).

And of course, you know, we would like to be on the road again. But we would like to keep doing this kind of stuff (Participant 30).

As *Zoom* mediated the performance via the various drawbacks of digital improvisation (latency, internet connection, editing out of sounds) players noted these elements of the Zoomesphere as an added emergent feature of the improvisation itself, an autonomous voice contributing to the improvisation.

Zoom is somehow almost like the final participant or the kind of the moderator of the improvisation…Zoom is like an additional player or conductor or something that’s choosing what we all hear (Participant 5).

Instead of highlighting the drawbacks of *Zoom*, many players spoke about working with these limitations:

We chose to embrace the technology and everything, all its faults and all its all its positives as well. It was like a journey of trying to explore how that could work for us (Participant 5).

[I] really enjoying some of the sort of affordances that the weirdness of the zoom scenario brought up. I mean, I like my approach to working with technology anyway, as you know, is like, I work with what it throws up to me. Like if my pitch tracker gives me funny notes, then I use that I’m never trying to like get it to accurately detect something. So having all those kinds of weird, things happen in zoom and the way that GIO like as a group kind of worked with those things (Participant 25).

I think only free improvisation can deal with…the limitations of the medium and….. I’ve seen in on Facebook I’ve seen a lot of people doing um little ensembles and so on they all seem really quite dull to me in not very exciting. I think that’s what Yeah, but this does seem like a proper life situation (Participant 27).

This resulted in a number of altered considerations on sound practice as described above in relation to volume and physicality, but also included practitioners re-considering what/how they chose instruments. In some cases, moving from the sonic to the visual as a new means for connection and creative communication.

I found myself using my voice a lot more and in ways that I normally wouldn’t have, and maybe it was sort of reaching through the computer screen…try and make a personal connection with somebody…in that kind of isolated sort of way…I also found myself getting more and more interested in, how you could use the visual side The process and seeing so many fantastic things that the other improvisers were doing…it’s about having fun as well (Participant 13).

If the audio content was unpredictable, players noted the usefulness of the visual contact in order to “fill in the gaps” where sound failed as an emergent listening practice. This “filling in” became, we suggest, like a new form of mental exercise and part of the new creative process, and in ways that pushed the improvisation into more overtly kinesthetic territory:

It’s almost like we were filling in the gaps, especially when we had the visuals to rely on. it almost felt like there was a bit of mental ventriloquism going on… the gestures we were making or the phrases we were trying to communicate still made sense. It was like the equivalent of having a few letters dropping out of a sentence. So you could still make sense of it. But not only just make sense of it and carry on, it just felt easier after several weeks as well (Participant 9).

### Re-imagining, Enhancing, and Augmenting Reality

The most notable results of this shift are practitioners’ acknowledgments of the welcoming atmosphere toward experimenting with transdisciplinary performance – “permission to play” (Participant 6).

Before I joined the session I don’t think I have confidence that I can enjoy the session. But I joined the session. The moment the first sound I made, George Burt smiled. Yes. I made first sound at the first time and George Burt smiled. And Maggie said some pleasant things for me. Yeah, it was very impressive for me. Yeah. And I thought maybe I can enjoy the session. And I think the GIO is a very good orchestra. So I am very glad to play with them (Participant 23).

This included experimentation with furnishing the digital space (via digital means or presenting objects/instruments/pets), costume/bodily presentation (makeup, suits, and gloves etc.) and embodied practices (dance, hand presentation, and facial expressions). These various factors became a transdisciplinary practice in the creation of virtual habitats, which existed synonymously and developed collaboratively through the groups willingness to experiment.

It was perhaps then not far to go from “filling in” the gaps to furnishing the boxes with visual and contextualizing materials and, over time, the individual participants increasingly made use of visuals, either visual backdrops, or the use of visuals to produce filmic effects on camera. Visual coding of still screenshots of sessions, always taken at moments when the highest number of participants were on the screen and comparing early screenshots to more recent sessions and noting whether or not each participant was employing visual materials (film, virtual backdrops, visual, or design features), suggests that the use of visual materials roughly doubled: from 29% on April 28 to 54% on September 15.

Over time, the visual component was heightened in what we have come to call the “theatre of the home’. Players can be seen altering their room/bodies/instruments via technologic inputs (*Zoom* virtual background, external video editing software), or physical alterations (fabric on camera, light experimentation). A number of new practices emerged as the ensemble experimented with the communicative possibilities of this new medium.

What’s been fascinating is finding objects, and finding things that I can use to create textures and stuff like that …. Yes, you can play with the fabrics and stuff that’s around (Participant 6).

Offices, living rooms, kitchens and gardens were deployed to create multiple contexts or “nows” that were drawn into the shared *Zoom* “room’. These “nows” can be understood to be in constant development as players exercised agency over the aesthetics of the space they wished to offer to the group. This created bespoke visual backdrops to perform with/in.

**FIGURE 3:**
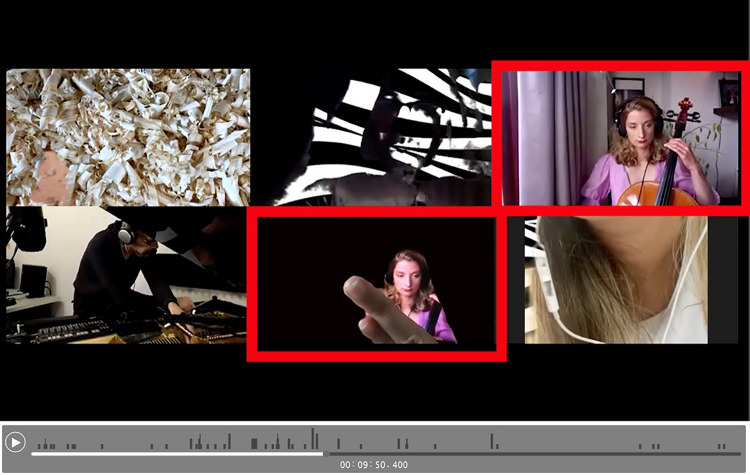
Player multiplied across “boxes.”

**FIGURE 4:**
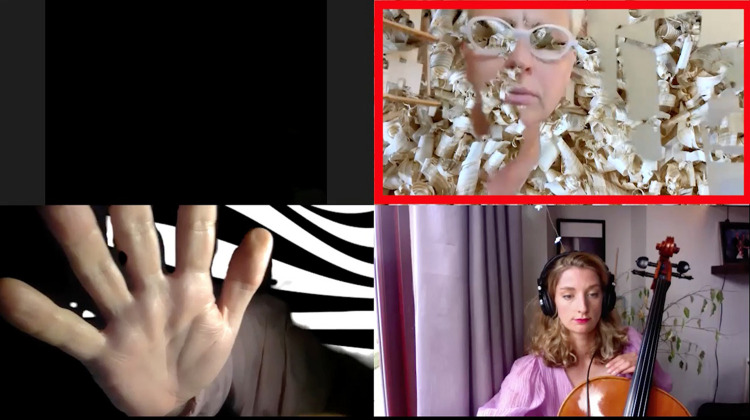
Player hybrid with virtual background.

**FIGURE 5:**
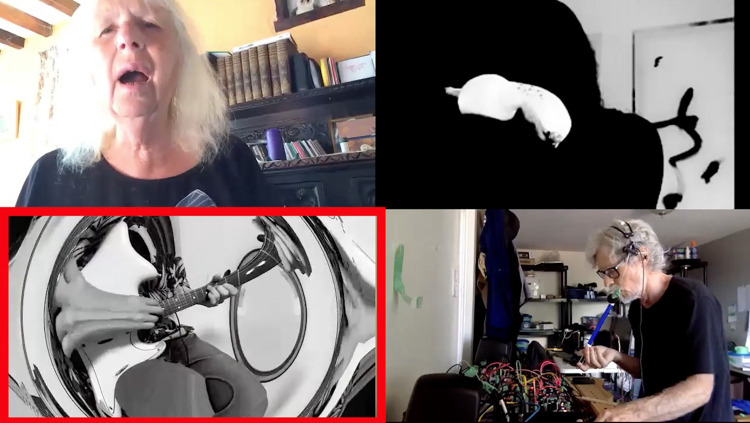
Player/instrument morph.

This visual play did not only include adaption of space, but also the alteration of presented self, perceived self and displayed embodiment. In some instances performers fade into a virtual background, warp instrument and body (Participant 7, small group Supplementary Video 2, 14’24”) or “welcome” other players into their space, in the small group Supplementary Video 2 at 9’47” we see the images of the other performers appearing in another players box. On the one hand this act was a poignant moment of modifying the isolation which was (and is) a reality of this context of being, during Covid-19, together apart. On the other hand, it was a creative and new strategy of sociability. Instances of the breadth of this experimentation are seen in Figure 3, player multiplication across ‘boxes’, Figure 4, player hybrid with virtual background, and Figure 5, player and instrument morph.

These are practical examples of experimentation with what a body can be when virtually enhanced. With *Zoom* as a new agent, ‘the” body gains ontological flexibility; it gains hybridity, contingency, possibility, and indeed new visually displayed freedoms, underscoring Lewis’ observation that:

‘…our bodies are not ourselves—not anymore. The network is the site of the production of knowledge, and the body animates that network. That makes living with creative machines an epistemological practice/project (Lewis, 2007).

In the practices and in the interview data we see participants engaging in what Kroker (2012) calls, “body drift,” in other words, the edges around what defines and separates bodies can be re-imagined. This re-imagining in turn permits and fosters new perceptions, including self-perceptions and, at times proprioception as bodies are re-configured sensorially through the virtual parameters and in ways that may produce the illusion of physical proximity and in ways that foreground virtual sensations and perceptions over “real” ones – and in ways that call into question the very dichotomy of “virtual’/’real” (DeNora, 2014, p. 119–120) and as the metamorphoses of identity, capacity, capability, ability, or skill shuttle between “virtual” and “real.” The Zoomesphere’s hybrid realm helps to expose such a lack of definable dichotomy, rather the “virtual” and the “real” are bound together, allowing reflective engagement with moment to moment drifts between physical and digital perceptions. As Participant 14 put it:

but there’s still this embodiment happening. I mean, there’s no embodiment, but it’s just like exactly what I mean one of the articles that I’m talking about in my installation, Nancy, you know, Jean-Luc Nancy, you know him? So he’s talking about this crying voice in the desert, that you hear a voice but where is the embodiment of the voice? If there is no embodiment, does the voice exists, isn’t it? Yeah, but I see this locations that you’re in. I see the environment that you’re in. I see like partial your hand there, I see a shadow. I see. I can imagine and completed in my head that I’m standing now where I’m sitting. I’m sitting on the left, but I’m here. But I’m still talking. But you don’t see me now. So am I here? Maybe it’s recorded. Maybe it’s not, I don’t know. And then you’re going back into your space that you are actually controlling. But you’re more aware of what’s going on now than you were before. Becauseyou’re always reflecting on yourself. So basically, you are always active. You’re never passive about what’s going on.

The virtual/real metamorphosis allowed players to try out new visual/virtual selves in ways that included contemplating new forms of self-presentation/being, one that when they eventually returned to physical venues might be sustainable there. This contemplation was associated with a sense of empowerment. The “theatre of home” afforded, in other words, a “safe space” in which to try out new selves – and to grow:

I’ve never used makeup in improvisers performances before…I thought this, this actually feels kind of safe. To try this. Cause it’s just, it’s just one thing in many…I didn’t overtly sort of share an artist statement about it or anything, but I felt like it was quite exhilarating for me to do that well, because I was, deconstructing a lot of, sort of worries that I have about. My visual as a woman in the music industry or in probably more in sound and kind of digital technology kind of profession that I’m in. You know, make up is something that, and color and clothes. It’s something that I love, but it’s something, I feel like I’m not really taken seriously if I wear too much of it or, or yeah well I have actually had comments about, you know, if I do my hair differently and things like that. So yeah. It was just, it was, you know, for me it felt quite kind of political and quite powerful, what I was doing it. I don’t know if that it really mattered to anyone else, but it was, yeah, it was quite helpful for me to sort of enact that and try that out. Over *Zoom*. Whereas if I did something like that in the CCA and just stepped into the middle of room and just started doing my makeup. I just, I don’t think I would have ever done that in the CCA. I think that just would have been too much or too frightening to do in a physical room. But who knows now afterward (Participant 9).

The Zoomesphere was associated with other forms of growth as well, including a kind of cognitive capacity or mode of attention, as Participant 19 explains:

And then in this virtual experience we become so real and maybe it was probably the efforts to go through this sympathy through this being together and try to focus on – not only on our presence, but on what we were doing together. Make a kind of expanded, I mean, the usual limits the frontiers of our, of our actions when we when we play together. I felt this in a very strong way…. Actually what I think was that sometimes, when we got into that dimension, it was like, like we are in a different brainwave, like we had this kind of, we have this internet thing here and we have something that’s connecting us even, I’m not playing the you know the mind power or…Actual I believe that improvisation can show us that we have a kind of capacity in our, in our cognitive and in our brain in our mind that actually we don’t use in a high level, we use in a very basic level as we’re dealing with this objective world. With the concrete world. And here we have the excuse to make it more powerful, I think (Participant 19).

**FIGURE 6:**
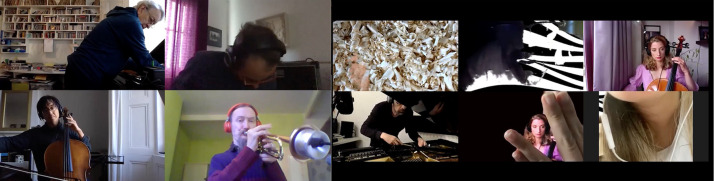
Comparison atmospheres.

As practices toward developing individual space continued, this resulted in various kinds of mini atmospheres. In the small groups (Supplementary Video 2) we see two such types of settings. At 5’21” there is a theatrical and augmented context, utilizing virtual backgrounds (shared across players), multiplying bodies on screen, and text based communication. This contrasts with the atmosphere of the group at 19’08” who use more traditional sonic signals in order to communicate. Figure 6 presented the comparison of these two distinct atmospheres.

**FIGURE 7:**
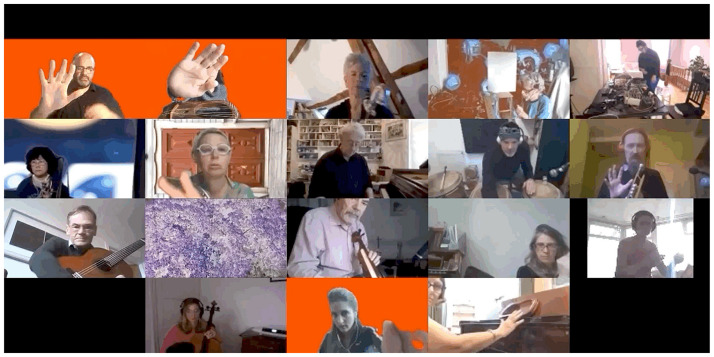
Imitation and emergent bodily themes 1. The Gif version, highlighting musicians’ movement, can be found in the Supplementary Material.

**FIGURE 8:**
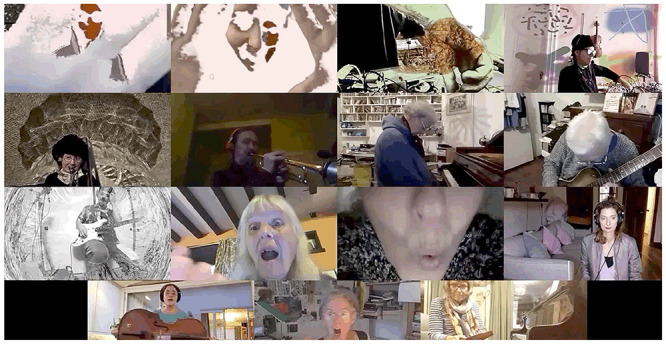
Imitation and emergent bodily themes 2. The Gif version, highlighting musicians’ movement, can be found in the Supplementary Material.

The notion of moving into the *Zoom* space does not imply that all members take up extra musical activities, but rather that the co-creation of different types of welcoming atmospheres (for small and large groups), creates a language which invites inclusion no matter what practice has been adopted. In the short excerpt (Supplementary Video 3), this language can be viewed as emergent as it infects, traveling around the group: hands and feet multiply across boxes synonymously with the musical gesture. Two such emergent bodily themes are presented in Figures 7, 8.

**FIGURE 9:**
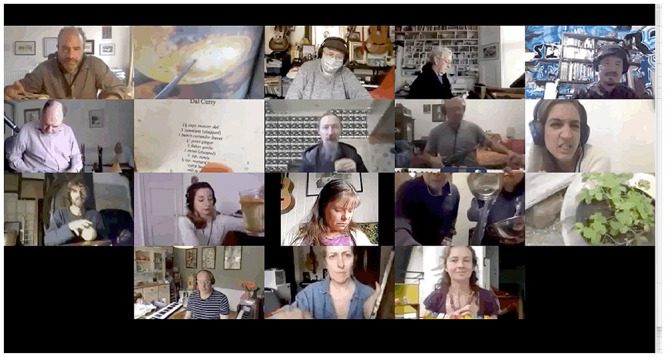
Showing objects/home/instruments 1. The Gif version, highlighting musicians’ movement, can be found in the Supplementary Material.

**FIGURE 10:**
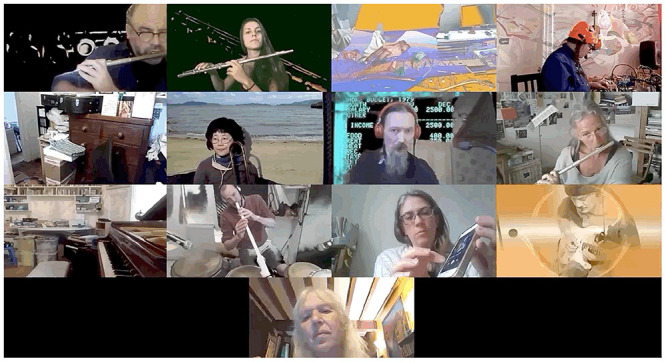
Showing object/home/instruments 2. The Gif version, highlighting musicians’ movement, can be found in the Supplementary Material.

Figures 9,10 reflect the environment of the Zoomesphere where players show objects, instruments, bodies, alongside music as an interdisciplinary narrative of community practice.

Moving into the Zoomesphere meant decorating individual *Zoom* space, as a new practice toward welcoming each other into these new intimate performance scenarios. This is a unique opportunity to watch improvisation do what it does so well: adapt. And more importantly adapt diversely and socially. As one player noted the skill was to “build relationships with strangers*”* (Participant 14)

When you’re given lemons you make lemonade, really…we were doing it all together, and you develop a kind of identity of working in a team, that goes beyond when you stop working (Participant 20).

In summary, the notion of the Zoomesphere emerged from the interviews as important in helping to inculcate a unique creative environment, what we have termed “The Theatre of Home’. This creative space, with its virtual, physical and mental components, functions as a shared and democratic environment, where participants can explore new ideas and collaborate, often leading to new creative insights. Participants describe this environment as safe, supportive and beneficial in helping them cope with the psychological demands of living under lockdown restrictions.

**FIGURE 11:**
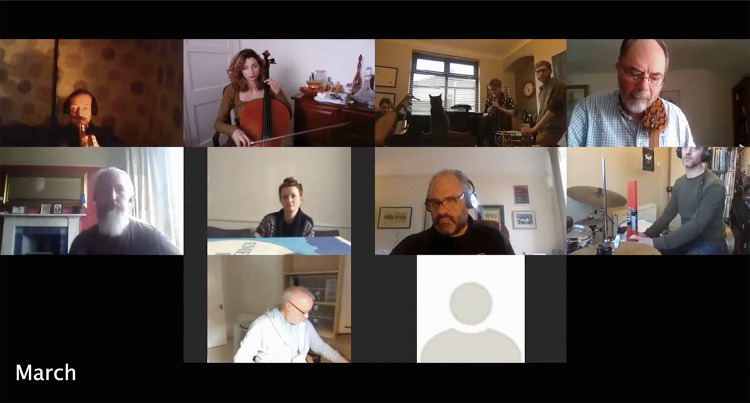
Time-lapse of rehearsals, all photos taken at moments when most participants were in attendance. The Gif version, highlighting musicians’ movement, can be found in the Supplementary Material.

Figure 11 presents a time-lapse of rehearsals developed from photographs taken at movements in each session when the most participants were in attendance.

## Discussion

The results highlight the multiple and complex ways in which online musical activities, and improvisational practices specifically, can be used to maintain and enhance social connections during periods of enforced physical distancing. These practices of “creative isolation” resonate with current debates around the potential for creative engagement in non-clinical contexts to enhance wellbeing (MacDonald, 2021b). The interviews also show how participants engage in what has been termed “health musicking” (Ruud, 2012, 2020) to create interdisciplinary narratives of community practice. “Health musicking” helps explain how musical engagement in everyday non-clinical contexts can provide a variety of benefits while highlighting there is no simple cause and effect relationship between music and health. Rather we can view music as a situated practice intertwined within a complex network of behaviors, emotions, and beliefs. It is this very complexity that lies at the heart of why music has potential to enhance wellbeing, since it is inextricably linked to notions of identity and community as well as creativity. The results therefore demonstrate a sophisticated and complex network of relationships where musical interactions facilitate psychological identity work leading to enhanced markers of wellbeing such as development in mood, positive emotions and reduced feelings of isolation. Participants frequently make explicit reference to multiple features as being important aspects of these positive effects. For example, the sophisticated way in which technological features merge with musical and social are clearly implicated by participants in explaining why music has beneficial effects. One salient feature is that the specifics of the Zoomesphere, as detailed above, facilitate new ways of being creative while maintaining and enhancing vital social bonds during periods of unprecedented global physical isolation.

Improvisational practices, with an emphasis on real time, spontaneous, collaborative interactions, are ideally suited to this nuanced interweaving of musical, psychological, social and technical features to facilitate health, as well as creative, benefits. Clift et al. (2010) suggest six emergent processes within music making (specifically singing but also prevalent in other forms of music making) that may contribute to these effects, which are also evident in the results presented above: attendant learning, positive emotions, focused concentration, controlled breathing, social bonding, education, and learning and active participation and we can clearly witness 5 of these generative processes in the results above.

Another key feature is the way in which participants report being supported in their endeavors. This is particularly evident for some of the players who were less experienced. This type of psychological support, termed scaffolding (Creech et al., 2014), has been shown to be particularly important when looking to enhance confidence and task orientated self-efficacy. The non-hierarchical, safe space as identified in the interviews allows for spontaneous decisions about the nature of individual creative inputs (starting, stopping, reflecting, introducing new ideas, responding, augmenting, and contrasting) and these types of decisions are clearly implicated in the reported results. These key categories of decision made by members of a group including; whether or not to change; whether to initiate or respond; and what kind of response to give have been identified as fundamental features of improvisational activity (MacDonald and Wilson, 2020). These choices allow improvisation at any level to become novel and valuable in particular ways where new discoveries are generated through the interplay of choices. Conceptualizing improvisation in this way can enhance our understanding of improvised interaction between performers from different disciplines or when using new technology. It is of note that other performance practices also moved to digital spaces. Similar improvising ensembles rehearsed using conference software’s^11^, virtual venues and residencies where set up^12^, live stream series occurred on various social media platforms^13^, and *Zoom* was also used for interactive live performance^14^. By contrast, GIO meetings between April and November did not involve live streamed performance and this non-audience environment contributed to participants’ understandings of GIO as a “safe space’. However, between November 26, 2020 and November 28, 2020 GIO presented, via live stream, its 13th annual international festival of improvisation titled Flattening The Curve which included all the participants in this study plus an extended group of United Kingdom and international artists^15^.

Framing improvisation as a moment to moment-decision based process helps explain how numerous unpredictable features can be integrated and utilized within the evolving and complex environment that is the focus of this article. Importantly, these decisions are not purely musical, but present in all types of improvising across artistic and social contexts (dance, theatre, turn taking in conversations, sporting activity, critical firefighting, and surgery etc.). They can also operate consciously and unconsciously. Thus, these types of decisions, which possibly lie at the heart of improvising, are accessible to everyone and allow for chance events and unforeseen outcomes, since the manner in which improvisatory decisions merge and develop can never be fully predicted. These external and performative elements are, as George Lewis notes, a practice of “real-time analysis, generation, manipulation, exchange, and transformation of meaning, mediated by (among other factors) the body, history, temporality, space, memory, intention, material culture, and diverse methodologies’ (Lewis, 2007). Moreover, these factors do seem to flourish in this digital realm particularly, which refers back to the group notion of actively engaging with – re-imaging realities toward creating a shared identity.

I think improvisation is the source of creativity for me. Improvisation is the most powerful, powerfully creative process there is because you know the mindfulness people meditators talk about being present, fully present, being in the moment, being able to cope with whatever arises. That’s what we’re doing. We’re actually fully immersed in each moment we’re responding to our own impulses, to what, to our environment, to other players, to other musicians. And it’s all happening as time unfolds. We’re literally in tune with the universe as it’s developing and unfolding. We’re right there at the cutting edge of life (Participant 6).

The hybridity (in all senses) of the “unfolding” experience in turn affords a kind of practical learning, its non-hierarchical features affording inclusion regardless of age, background, or expertise. This spirit, very much linked to existing histories of the Feminist Improvising Group (FIG) highlights what Maggie Nicols calls, ‘social virtuosity’. Indeed, it has been argued that conventional notions of musical virtuosity are too narrow, over emphasizing technical mastery, and new virtuosities which could include virtuosic listening, collaborating and decision making, may be universally accessible and fundamental to improvising (MacDonald and Wilson, 2020). Thus, rather than impeding socially motivated musical techniques, the digital nature of the meeting space actually bolstered and inspired new possibilities for co-developing a technological community and, as such, has enhanced the improvisatory skills, repertoire and cultural “toolkit” of the participants. Willingness, acceptance, and spontaneous receptibility have assisted in the evolution of this environment, ultimately encouraging experimentation, self and group discovery and an overarching feeling of safety for a newly established artistic community in a time of great unrest. It remains to be seen the extent to which some of these skills are transferred to settings outside of GIO, where “social improvisation” can be helpful – as for example in online interactions linked to work, conflict resolution, social services, and daily life.

## Conclusion

Music can be characterized as accessible, creative, universal, collaborative, communicative, and emotional. One of its primary functions is to enable communication, and improvisation as a constituent process, helps establish music as a distinct and complex channel of communication. It is deployed in countless situations to facilitate contact between people, to help maintain community through the sharing of ideas, mutual creative engagement and provide a reason to be in contact with others, so vital during times of enforced isolation. This article takes one specific example and highlights numerous ways in which improvisation can facilitate health improvements and act as a mechanism for changes of consciousness while integrating new ways of collaborating: a virtual tribe. Innovative group based creative practices can be undertaken in the domestic environment, creating what we have termed “The Theatre of Home” and demonstrating how online synchronous improvisational activities can enhance community, create novel improvisatory techniques and new experiences in creativity when physical distancing is mandatory.

## Data Availability Statement

The datasets presented in this article are not readily available because full dataset only available to authors. Requests to access the datasets should be directed to corresponding author (raymond.macdonald@ed.ac.uk).

## Ethics Statement

The studies involving human participants were reviewed and approved by Edinburgh College of Art, The University of Edinburgh, Ethics Committee. The patients/participants provided their written informed consent to participate in this study. Written informed consent was obtained from the individual(s) for the publication of any potentially identifiable images or data included in this article.

## Author Contributions

All authors listed have made a substantial, direct and intellectual contribution to the work, and approved it for publication.

## Conflict of Interest

The authors declare that the research was conducted in the absence of any commercial or financial relationships that could be construed as a potential conflict of interest.

## Footnotes

1 COVID Social Study: https://www.covidsocialstudy.org/results

2 COVID Minds: https://www.covidminds.org/longitudinal-studies

3 https://www.theguardian.com/world/2020/mar/14/solidarity-balcony-singing-spreads-across-italy-during-lockdown

4 https://www.dailyrecord.co.uk/entertainment/celebrity/scots-dj-george-bowie-pledges-21723745

5 https://www.bbc.com/news/av/uk-52860785

6 https://www.glasgowimprovisersorchestra.com/giofest/programme/

7 https://www.glasgowimprovisersorchestra.com/press/

8 https://research.hud.ac.uk/institutes-centres/irimas/, ERC grant agreement no. 741904

9 https://www.jacktrip.org/

10 https://www.jamkazam.com/

11 Toronto Improvising Orchestra (CA), Oxford Improvising Orchestra (United Kingdom), The Gathering (United Kingdom)

12 Chez Roger (CZ), now closed due to “re-opening,” Serge and Neverthere Festival (United Kingdom)

13 JeffersonParkEXP, Open Improvisation Series: Online Edition, AMPLIFY 2020: quarantine

14 Mamoru Iriguchi Zoom Dark Mode

15 https://www.glasgowimprovisersorchestra.com/
